# Electroencephalographic Microstates in Schizophrenia and Bipolar Disorder

**DOI:** 10.3389/fpsyt.2021.638722

**Published:** 2021-02-26

**Authors:** Fanglan Wang, Khamlesh Hujjaree, Xiaoping Wang

**Affiliations:** Department of Psychiatry, National Clinical Research Center for Mental Disorders, The Second Xiangya Hospital of Central South University, Changsha, China

**Keywords:** electroencephalographic microstate, resting state, schizophrenia, bipolar disorder, resting-state networks

## Abstract

Schizophrenia (SCH) and bipolar disorder (BD) are characterized by many types of symptoms, damaged cognitive function, and abnormal brain connections. The microstates are considered to be the cornerstones of the mental states shown in EEG data. In our study, we investigated the use of microstates as biomarkers to distinguish patients with bipolar disorder from those with schizophrenia by analyzing EEG data measured in an eyes-closed resting state. The purpose of this article is to provide an electron directional physiological explanation for the observed brain dysfunction of schizophrenia and bipolar disorder patients.

**Methods:** We used microstate resting EEG data to explore group differences in the duration, coverage, occurrence, and transition probability of 4 microstate maps among 20 SCH patients, 26 BD patients, and 35 healthy controls (HCs).

**Results:** Microstate analysis revealed 4 microstates (A–D) in global clustering across SCH patients, BD patients, and HCs. The samples were chosen to be matched. We found the greater presence of microstate B in BD patients, and the less presence of microstate class A and B, the greater presence of microstate class C, and less presence of D in SCH patients. Besides, a greater frequent switching between microstates A and B and between microstates B and A in BD patients than in SCH patients and HCs and less frequent switching between microstates C and D and between microstates D and C in BD patients compared with SCH patients.

**Conclusion:** We found abnormal features of microstate A, B in BD patients and abnormal features of microstate A, B, C, and D in SCH patients. These features may indicate the potential abnormalities of SCH patients and BD patients in distributing neural resources and influencing opportune transitions between different states of activity.

## Introduction

Bipolar disorder (BD) and schizophrenia (SCH) are mental illnesses that share a few clinical manifestations, such as hallucinations and delusions (1, 2). Numerous researchers have questioned whether SCH and BD constitute 2 separate mental disorders due to their overlapping features (3–5). Euthymic patients with bipolar disorder still perform poorly on cognitive measures, which means that the impairment is not just limited to periods and residual stage of disease (6–9). The neurocognitive dysfunction that accompanies SCH is enduring (10, 11). Recent studies have found that a large number of BD patients have neurocognitive dysfunction too (12, 13). In contrast to BD, it is widely known that significant dysfunction is much more unique to SCH. For both SCH and BD, multiple candidate endophenotypes have been suggested (14, 15). This potential endophenotype for SCH is visual backward masking (VBM) (16) particularly the shine-through approach, and has a far greater sensitivity than most of the other cognitive and perceptual processes (17). A previous study indicated that visual backward masking deficiency is not unique to schizophrenia, but a larger range of functional psychosis. Both SCH and BD may suffer insufficient objective strengthening (18). A recent study showed that particular neurological functions can distinguish SCH from BD and also indicate a presumptive endogenous phenotype that can distinguish the genetic susceptibility of SCH from another severe psychological disease (19).

In SCH patients, key adjustments have mainly been noticed throughout the interrelationship of the medial pre-frontal cortex (mPFC) as well as anterior cingulated cortex also with the limbic striatum; researchers have also identified homogenization of the default mode network (DMN) of SCH about specific microstate dynamics (20). Intriguingly, in BD patients the irregular functional structure and degree of activity of the brain network during the rest period may be caused by maladjustment of neurotransmitter activity, such as that of serotonin and dopamine (21). The complex connection between the mPFC as well as the posterior cingulate cortex (PCC) changes in BD patients, and it tends to change less as time goes on (22). A recent meta-analysis suggests that the dorsolateral pre-frontal cortex (DLPFC) plays a special role in SCH (23, 24). The instability of the DLPFC was proposed to be a central characteristic of SCH (25, 26). Utilizing brain scanning technology, the latest research findings have contributed to major conceptual changes in the perception of higher brain function and how specific brain pathologies impact those functions (27). Additionally, the understanding of the resting state of the brain has undergone a fundamental change: the mainstream assumption is that the brain does not just keep inactive until a new stimulus triggers a response but is naturally active in an organized manner during rest, which is the best preparation for processing the stimulus (28–30).

In our study, we investigated the use of microstates to distinguish BD patients from the SCH patients by analyzing their EEG data measured in an eyes-closed resting state. EEG can record fluctuations with a time scale, so EEG is more suitable for studying the time dynamics and effects of the resting state and their impact on stimulus processing (27). The microstates are considered to be the cornerstones of the mental states shown in EEG data (31, 32). Studies showed that microstate analysis can help reveal the importance of the modularity of brain dynamics and their function in behavioral control, as well as the characteristics of the cerebral disease (31, 33). Microstate analysis is increasingly recognized as an innovative method offering straight-forward characterizations of brain-states. Its use for understanding brain function has been proven in healthy people (31) and clinical patients (34). Microstate analysis can be a very useful method for exploring the brain network functions of patients with mental illness (35). Scholars are currently investigating the potential applications of electroencephalograms (EEGs), and the characteristics of scalp EEG are commonly applied to distinguish between BD and SCH (36).

Previous studies suggested that human microstates may be correlated with particular resting-state networks found in functional magnetic resonance imaging (fMRI) studies due to the association between the presence of microstates and particular resting-state network actions (37–39). There are 4 common map modes for microstates, which are labeled A, B, C, and D. Synchronized EEG and functional magnetic resonance imaging indicated a correlation of these microstates with all kinds of neural networks, including audition (microstate A), vision (microstate B), saliency (microstate C), and frontal-apical network (microstate D) (37, 38). Microstate abnormalities in SCH patients could indicate a malfunction of regular network functions underlying medical pathogenesis since EEG microstates represent the organized neural activity groups of the cerebrum. Microstate modifications in SCH, therefore, tend to indicate impaired coordination, reduced functional structure, or elevated disturbance in brain functions, which could be the neurophysiological basis for the symptoms of SCH (34). The multiple clinical symptoms and cognitive impairment of SCH have long been interpreted as the dysfunction of extensive neural systems instead of dysfunction in a particular brain area (40, 41). Many studies of SCH patients found abnormal temporal dynamics of EEG microstates compared with HCs (27, 42). Microstate abnormalities are frequently reported in SCH (34, 43–46). These abnormalities included the less duration of 2 microstate classes (B and D) (34, 46–48) and the greater occurrence of another microstate (class A) (34, 46, 47) compared with HCs. The main reliable results are greater Class C and less Class D in SCH, referring to 2 meta-analyses (49, 50).

A large number of research studies have shown that the EEG microstates in patients with neuropsychiatric disorders are changed (27, 42); however, while microstate analysis has the potential to detect global brain dynamic damage, microstates have not been fully developed in BD. To date, only 2 studies have studied microstate EEG in BD patients (51, 52).

We need to understand more of the underlying pathophysiology to determine objective biomarkers for SCH patients and BD patients, which would be useful for improving patient diagnosis and treatment stratification. Similar to BD, the widely distributed neural circuits appear to be changed in SCH, mainly impacting the frontal, temporal, and subcortical structures (53). Therefore, the study of EEG microstates could offer a novel method to clarify the neurophysiological basis of SCH and BD. The purpose of this article is to provide an electro-directional physiological explanation for the observed brain dysfunction of SCH and BD. Is there any mutual mechanism for these serious neuropsychiatric diseases? How can the findings help to classify these 2 neuropsychiatric disorders? This study aims to evaluate the microstate characteristics of EEG in patients with SCH and BD and HCs. Despite active research using microstates, as far as we know, no research-based on microstates that aims to distinguish between BD and SCH has ever been conducted. We hypothesized that greater presence of microstate A and less presence of microstate B in BD patients and a greater presence of microstate C and less presence of microstate D in SCH patients based on larger evidence.

## Materials and Methods

### Subjects

Twenty SCH patients (mean age 25.2 ± 6.8, 15 female), 34 patients with BD (mean age 22.8 ± 4.12, 13 female), and 35 HCs (mean age 24.9 ± 6.2, 25 female) took part in our experiment. All subjects were older than 18 years old and younger than 40. Fifteen SCH patients took at least 15 mg olanzapine with or without combining another antipsychotic drug per day, other patients took paliperidone 9 mg per day or risperidone 6 mg per day or amisulpride 0.6 g per day or ziprasidone 120mg combining with aripiprazole 10 mg per day or aripiprazole 20 mg. Furthermore, only two SCH patients took antipsychotic drugs combining with mood stabilizers, such as 900 or 600 mg lithium carbonate. Twenty six BD patients took mood stabilizers (lithium carbonate or sodium valproate or Magnesium Valproate or lamotrigine) combining with second-generation antipsychotic drugs (olanzapine or seroquel or aripiprazole or paliperidone or risperidone). Additionally, all BD patients were bipolar I and had a history of psychotic symptoms. All the patients were dexterous. The Second Xiangya Hospital of Central South University approved our experimental proposal, and professionals who were trained by the psychiatry department acquired handwritten informed consent from every participant. We carried out this research according to the recommendations of the Ethics Committee for Human Research of Second Xiangya Hospital of Central South University.

A psychiatrist acquired the demographic information and psychiatric history of the subjects through face-to-face interviews. We used ICD-10 and DSM-5 criteria as well as the MINI-International Neuropsychiatric Interview to diagnose the SCH patients and patients with BD. We also used the Montgomery-Asberg Depression Rating Scale (HAMD) and Young Mania Rating Scale (YMRS) to evaluate depressed and manic symptoms. We excluded subjects using the following criteria: a history of head injury, history of drug abuse, epilepsy history.

### EEG Data Collection and Processing

We recorded 3 min of resting-state EEG data as participants closed their eyes and sat on a chair comfortably. We obtained EEG data using a 64-channel system (Brain Product). EEG data were recorded and sampled at 5,000 Hz. We kept the impedances under 5 Kohm. We preprocessed the data using Matlab 2013b and the MATLAB EEGLAB toolbox (54). A 48–52 Hz Parks-McClellan stop-band Notch-filter was used to remove electric-interference from the 50 Hz-line. Then the data were band-pass filtered (0.1–40 Hz) and downsampled to 250 Hz. We segmented the data into epochs of 2 s and we rejected bad epochs with obvious muscle activity when we checked visually. Besides, we used independent component analysis (ICA) to remove the oculomotor component when necessary. Then, we referenced the data to the average reference, with a bandpass filtered to 2–20 Hz.

### Microstate Analysis

**Figure 1:**
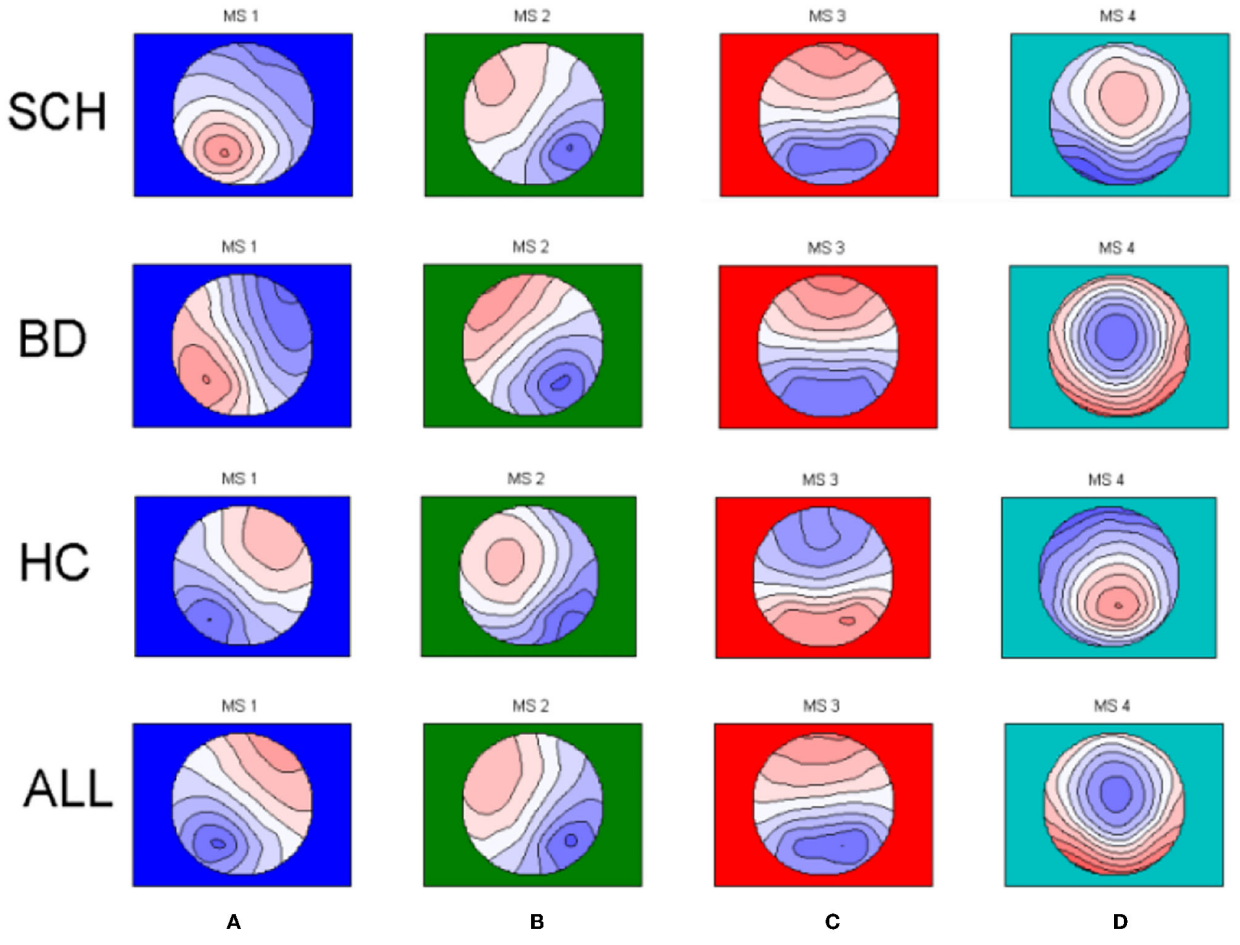
The four microstate topographies **(A–D)** identified in the group clusterings across schizophrenia patients (SCH) and bipolar patients (BD) and healthy controls (HC) and in the global clustering across all subjects (SCH+BD+HC).

Microstate analysis was performed using Matlab 2013b. The 4 microstate classes for SCH patients, patients with BD, and HCs are shown in Figure 1. The 4 microstate classes explained 76.2, 74.1, and 76.0% of the global variance in SCH patients, BD patients, and HCs, respectively, and one-way ANOVA showed no significant differences (*p* = 0.126) among SCH patients, BD patients, and HCs. To eliminate the influence of the difference in the template maps in different epochs, we calculated the global field power (GFP) of the EEG data with the longest duration of each subject. All GFP peaks were from all participants clustered in the first steps to get the template maps, and then all GFP peaks from the first step themselves clustered in a second step to determine which class of the template maps at each time point of each subject belongs to. The modified k-means algorithm was used and limit k−100 when clustering.

The EEG data were divided into 4 types of microstate topographies to keep consistency with previous studies. The purpose of this research is to investigate the four EEG microstates, we obtain four conventional microstates for three different groups and as well for all participants regardless of patient status (Figure 1). Global segmentation was done across groups and one set of template maps was fitted to the original data. These four resulting all group template maps (Figure 1) were further used to extract the microstate characteristics.

Based on (55) the microstate transition was calculated, which is the probability of transition between every 2 microstates back and forth. Twelve observed possible transitions were considered.

## Results

According to the results in Table 1, one-way ANOVA showed no significant differences in educational years, age, and duration of the disease (*p* = 0.686, *p* = 0.286, and *p* = 0.960, respectively) among SCH patients, BD patients, and HCs. The chi-square test showed no significant difference in gender (*p* = 0.129) among SCH patients, BD patients, and HCs. One-way ANOVA showed that there was no significant difference in the BPRS total score, PANSS total score, MADRS total score, or YMRS total score (*p* = 0.492, *p* = 0.269, *p* = 0.397, and *p* = 0.807, respectively), between SCH patients and BD patients. If *p* was smaller than 0.05, then we considered the findings to be significant.

**Table 1:** Subject features.

**Variables**	**SCH**	**BD**	**HC**	**Statistics**	***P*-value**
Categorical variables: (*N*)				Chi-square test	
Gender (*n*, female/male)	5/15	13/13	10/25		0.129
Continuous variables: mean (SD)				ANOVA/F	
Educational years	14.1 ± 3.3	14.4 ± 2.2	13.8 ± 2.5	0.379	0.686
Age	25.2 ± 6.8	22.8 ± 4.12	24.9 ± 6.2	1.273	0.286
Duration of disease	5.4 ± 4.5	5.5 ± 3.7		0.003	0.960
BPRS total score	36.3 ± 9.5	34.5 ± 7.3		0.480	0.492
PANSS total score	52.4 ± 12.7	48.6 ± 10.2		1.253	0.269
MADRS total score	9.8 ± 6.5	12.0 ± 10.0		0.730	0.397
YMRS total score	8.3 ± 5.6	8.8 ± 8.1		0.060	0.807

The details of the time characteristics of 4 microstates are summarized in Table 2. Duration (in s) was the mean time that a certain microstate continuously presented. Coverage (in %) was the centage of the cumulative time that a certain microstate took. The occurrence was the average times that a certain microstate was appearing per second. We used repeated-measures ANOVA and simple effects tests for the analysis of mean occurrence per second, coverage, and microstate duration, with microstate (A, B, C, and D) as the within-subject factor and group (SCH patients, BD patients, and HCs) as the between-subject factor. We used one-way ANOVA and Bonferroni tests for the analysis of transition probabilities. For the duration, we found a significant main effect of microstate [*F* = 23.891; *p* < 0.001], and no significant microstate ^*^ group interaction effect [*F* = 1.612; *p* = 0.147], but a significant main effect of group [*F* = 3.686; *p* = 0.030]. For the mean occurrence per second, we found a significant main effect of microstate [*F* = 30.955; *p* < 0.001], as well as a significant microstate ^*^ group interaction effect [*F* = 3.926; *p* = 0.001], and there was a significant main effect of group [*F* = 6.405; *p* = 0.003]. For the coverage, we found a significant main effect of microstate [*F* = 28.505; *p* < 0.001] and a significant microstate ^*^ group interaction effect [*F* = 3.076; *p* = 0.007], but there was no significant main effect of group [*F* = 0.019; *p* = 0.981].

**Table 2:** Microstate parameters.

**Microstate**	**A**	**B**	**C**	**D**
**Duration (s)**				
Schizophrenia (mean ± s.d.)	6.79E-2 ± 7.3E-3	6.48E-2 ± 5.5E-3	9.27E-2 ± 1.93E-2	6.53E-2 ± 1.00E-2
Bipolar (mean ± s.d.)	6.48E-2 ± 8.1E-3	6.37E-2 ± 7.6E-3	7.75E-2 ± 2.52E-2	5.87E-2 ± 1.08E-2
Controls (mean ± s.d.)	6.56E-2 ± 9.1E-3	6.29E-2 ± 6.9E-3	7.99E-2 ± 2.16E-2	6.39E-2 ± 1.07E-2
**Occurrence (s**^**−1**^**)**				
Schizophrenia (mean ± s.d.)	3.19 ± 6.8E-1	3.00 ± 5.4E-1	4.19 ± 4.2E-1	3.26 ± 6.1E-1
Bipolar (mean ± s.d.)	3.74 ± 6.8E-1	3.97 ± 8.1E-1	4.20 ± 5.2E-1	3.50 ± 6.0E-1
Controls (mean ± s.d.)	3.62 ± 6.1E-1	3.41 ± 7.4E-1	4.20 ± 5.4E-1	3.65 ± 7.4E-1
**Coverage (%)**				
Schizophrenia (mean ± s.d.)	2.17E-1 ± 5.6E-2	1.94E-1 ± 4.2E-2	3.75E-1 ± 8.8E-2	2.13E-1 ± 5.9E-2
Bipolar (mean ± s.d.)	2.39E-1 ± 4.8E-2	2.49E-1 ± 5.4E-2	3.07E-1 ± 7.4E-2	2.03E-1 ± 5.1E-2
Controls (mean ± s.d.)	2.35E-1 ± 5.1E-2	2.13E-1 ± 5.2E-2	3.22E-1 ± 8.1E-2	2.29E-1 ± 5.7E-2

**Figure 2:**
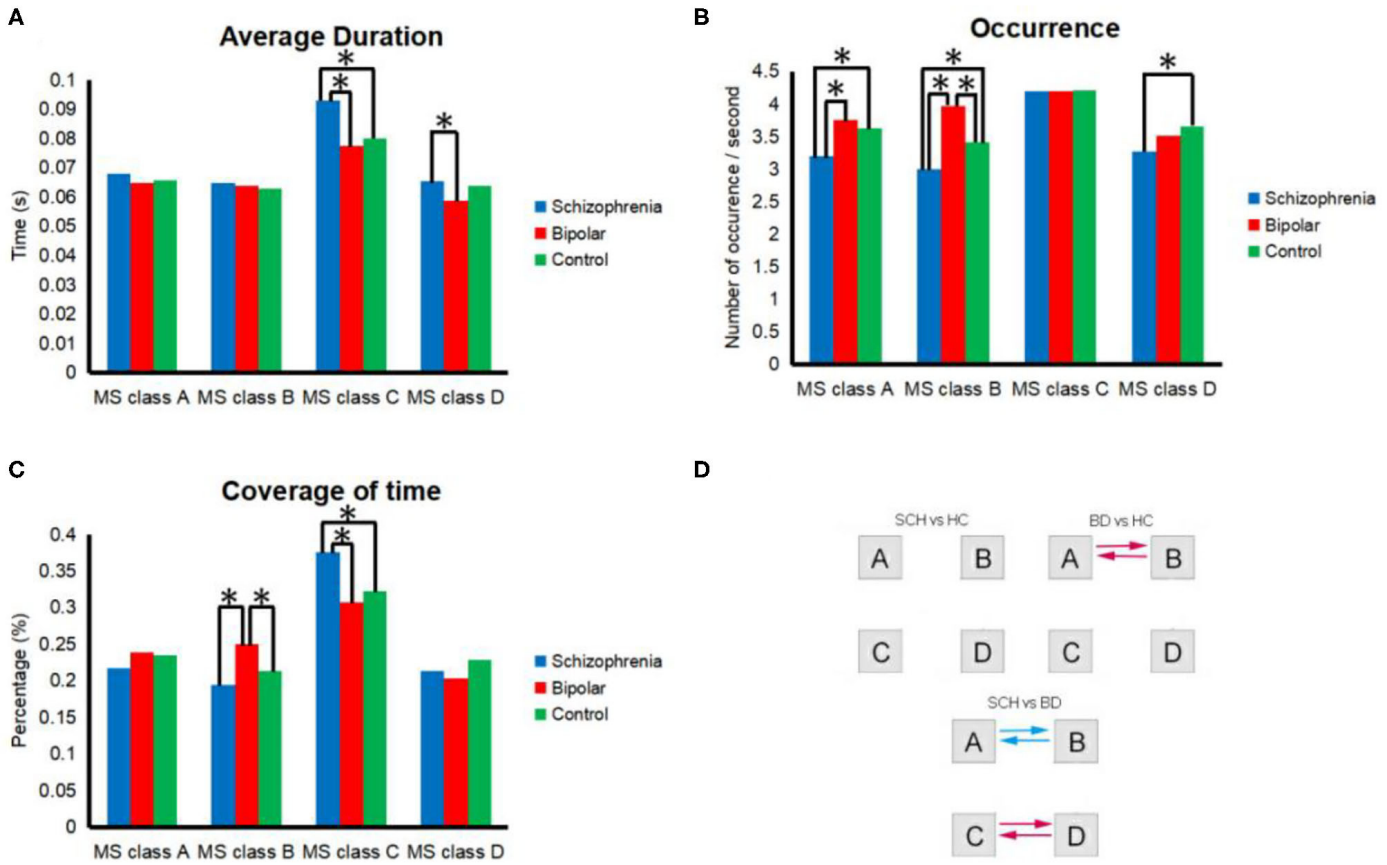
Properties of microstates in general. All of the 4 microstate classes (MS class) are presented in each segment. **(A)** occurrence per second; **(B)** total time coverage; **(C)** average duration; **(D)** transition probability. Transition probabilities were analyzed between microstates for HCs and SCH patients, HCs and BD patients, or BD patients and SCH patients. Red and blue indicate significant differences between HCs and BD patients or between BD patients and SCH patients. Specifically, red indicates a higher probability among SCH patients than BD or a higher probability among BD than HCs, and blue indicates a lower probability. *Means significant difference between two groups.

As shown in Figure 2, we found 10 features that showed a significant difference between SCH patients and BD patients and five features that showed a significant difference between SCH patients and HCs. We also found four features that showed a significant difference between BD patients and healthy controls. According to the results of *post hoc* comparisons, the occurrence of microstate class A and B (*p* = 0.006; *p* < 0.001) and the coverage of microstate class B (*p* < 0.001) were significantly smaller, while the duration of microstate class C and D (*p* = 0.024; *p* = 0.039) and the coverage of C (*p* = 0.006) were significantly greater in SCH patients compared with BD patients. Also, the occurrence of microstate class A, B, and D (*p* = 0.024; *p* = 0.045; *p* = 0.040) were significantly smaller, while the duration and coverage of the microstate class C (*p* = 0.043; *p* = 0.023) were significantly increased in SCH patients compared with HCs. The occurrence and the coverage of microstate class B (*p* = 0.004; *p* = 0.007) were significantly increased in BD patients compared with HCs.

The transition probability from microstate class A to B (*p* = 0.002) and from microstate class B to A (*p* = 0.001) was significantly smaller in SCH patients compared with BD patients. The transition probability from microstate class C to D (*p* = 0.019) and from microstate class D to C (*p* = 0.021) was significantly greater in patients with SCH compared with BD. The transition probability from microstate class A to B (*p* = 0.011), from microstate class B to A (*p* = 0.007) was significantly greater in BD patients compared with HCs.

## Discussion

We showed that microstate segmentation of the resting-state EEG recordings provides useful features that successfully distinguish SCH patients and BD patients and HCs. Several studies have unanimously confirmed the abnormal time dynamics of the EEG microstates in SCH patients (34, 44–46, 48, 56–58); however, less focus has been given to BD regarding the microstate resting EEG. As far as we know, there are no research studies that have tried to distinguish SCH patients from BD patients using microstate analysis. In our study, SCH patients did not differ significantly from BD patients in terms of depression, mania, or psychiatric symptoms. Despite this fact, SCH patients showed a significantly greater presence of microstate classes C and D significantly smaller presence of microstate classes A and B compared with BD patients.

We found that the occurrence of microstate class A was significantly less than in SCH patients compared with BD patients and HCs. For SCH patients, research studies have reported greater occurrence (47, 59) and coverage of microstate A (34, 59). For euthymic BD patients, previous research found increased occurrence and coverage of microstate A (60). For SCH patients, changes of microstate class A were correlated with auditory hallucinations (43). Previous fMRI-EEG research has shown that microstate A was mainly related to the negative blood-oxygen-level dependent activation of the mid temporal gyrus and bilateral superior temporal gyrus, which are related to speech processing (37). In addition, recent studies have estimated the sources of EEG microstates. The sources of microstate A are located on the left side of the occipital gyri, insula, temporal lobe, and medial pre-frontal cortex (mPFC) (61). Previous fMRI-EEG research reported the microstate A was correlated with the auditory network (37). Moreover, an fMRI study also found resting-state functional connectivity abnormality of the insula (62), the auditory network (63), and the medial pre-frontal cortex (64) in BD patients. The abnormality of the medial pre-frontal cortex was as well-reported as an important shared abnormality in BD patients and SCH patients (65). Previous studies found the default mode network from the left posterior cingulate cortex to the bilateral mPFC and bilateral precuneus has low connectivity, and significant connectivity of the left subgenual anterior cingulate cortex to the right inferior temporal gyrus also reduced (64) in BD patients.

We found the occurrence and coverage of microstate B were significantly greater in BD patients compared with SCH patients and HCs. Furthermore, the occurrence of class B was as well-significantly less in SCH patients compared with HCs. Previous studies reported decreased (59) occurrences and decreased (59) coverages of microstate B in SCH patients. Previous studies report a reduced presence of microstate B in Euthymic BD patients compared to HC (66). Microstate B was related to the visual network, according to previous studies (37, 61, 67), and imagination associated with the awareness of situational personal memory, that is, the mental visualization of the situation (68). Compared with the special case of mental arithmetic operations (mathematical tasks, unrelated to self), greater microscopic state B was found in participants who were asked to retrieve past events related to self in fMRI-EEG records (69). Microstate B was correlated with the mental visualization of a situation (69). A recent study reported that a greater presence of microstate B was correlated with more severe anxiety (60).

The duration and coverage of microstate class C were significantly greater in SCH patients compared with HCs and BD patients. A previous article found that C microstates appeared more and D microstates appeared less in schizophrenia patients compared with controls (49). Some articles found that the occurrence of class C was significantly greater in schizophrenia patients compared with healthy controls (34, 45, 47, 56). Microstate C was positively associated with the activation of blood oxygen level-dependent (BOLD) in the bilateral inferior frontal gyri, the posterior part of the anterior cingulate cortex, and the right anterior insula (37). The activation of microstate C was derived from the bilateral part of the medial temporal gyrus and the lateral part of the parietal lobe. We assigned these areas to the self-experience subsystem according to the fMRI results (69). Moreover, microstate class C was considered to mirror the activation of the default mode network (DMN) (27, 37, 70). Furthermore, DMN is important for self-focusing and self-referencing processing. Therefore, our results might indicate the abnormality of situational self-memory and abnormal self-experience in SCH patients.

We found the occurrence of microstate class D was significantly less in SCH patients compared with HCs. Also, the duration of microstate D was significantly greater in SCH patients than BD patients. Tomescu et al. (56) found that the occurrence of class D was significantly decreased in schizophrenia patients compared with healthy controls, while recent studies found that the occurrence of class D was significantly increased in schizophrenia patients compared with healthy controls (59). According to the dorsolateral pre-frontal cortex (DLPFC) seem to have a high pathogenic value in SCH (23, 71), and previous researches showed that the frontoparietal network was involved in SCH (72, 73), the reduction of the microstate D clearly showed that the function of the network was impaired (47). Previously, microstate D was thought to be related to the attention/cognitive control network consisting of the frontoparietal region (37, 61), and the source of microstate D was thought to belong in the region of the frontoparietal control network (FPCN) (74). For SCH patients, the observed increase in microstate C and decrease in microstate D involved abnormal activation of the frontal-parietal control network (FPCN) and default mode network (DMN) during externally directed and self-directed cognition.

In our study, the transition probability analysis revealed a greater frequent switching between microstates A and B and between microstates B and A in BD patients than in SCH patients and HCs. Besides, we found a less frequent switching between microstates C and D and between microstates D and C in BD patients compared with SCH patients. Previous studies that compared schizophrenia and other diseases indicated that the imbalance of C and D states might be unique to SCH patients (27). The change in transition probability between microstate classes may lead to insufficient activation of the network therein and may cause abnormal functions of more than 1 network. Previous magnetic resonance imaging research showed that a more coherent DMN in temporal regions and the superior frontal gyrus appeared more frequently in SCH patients than in BD patients and HCs. In addition, a more coherent DMN in the insula appeared more frequently in BD patients than in SCH patients and HCs (65). Our results suggested that BD affects the auditory and visual sensory network instead of the higher-order (salience, central executive) functional networks.

Ellen et al. found functional hyperconnectivity of the default mode network in SCH patients (75). Compared with BD patients, SCH patients exhibited decreased structural aggregation in the forehead region (76). In particular, compared with BD patients, SCH patients had higher nodal aggregation coefficients in the left inferior frontal cortex and the left ascending ramus of the lateral sulcus (77). The connection intensity of SCH patients was lower than that among BD patients and HCs, but there was no difference in network topology between groups. In contrast, BD patients in another study had a less complete network topology, while the connection intensity was not disturbed (78). Recent studies have found that the connectivity of the whole brain network was increased in SCH patients but not in BD patients. This revealed that a highly synchronized basic state is widespread, which may hinder the cognitive ability of the disease (79). All in all, the results from fMRI and EEG research studies have suggested that both low and high connections exist in patients and showed complex changes in the functional static network. Our findings of a greater presence of microstate B in BD patients might be related to the greater connection strength of the visual sensory function network, while the greater presence of microstate C in SCH could be associated with the enhanced connection strength of the relevant networks that involve the posterior part of the anterior cingulate cortex as well as the bilateral inferior frontal gyri, the right anterior insula, and the left claustrum.

In conclusion, this study represents the first attempt to compare EEG microstates between SCH patients and those with BD using EEG data collected during an eyes-closed resting state. Our results demonstrate the greater presence of microstate class B in BD patients, and less presence of microstate class A and B, a greater presence of microstate class C, and less presence of D in SCH patients compared with BD. In addition, a greater frequent switching between microstates A and B and between microstates B and A in BD patients than in SCH patients and HCs and less frequent switching between microstates C and D and between microstates D and C in BD patients compared with SCH patients. Therefore, in clinical practice, microstate analysis can play an important role in diagnosing patients more accurately and treating them properly. Furthermore, EEG microstate analysis, which is a creative and feasible method, can be used as an index to help identify different aspects of pathogenesis between SCH patients and BD patients.

## Limitation

All the patients were medicated and the sample sizes are small and the results might not be representative because both SCH and BD are very heterogeneous.

## Data Availability Statement

The original contributions presented in the study are included in the article/supplementary material, further inquiries can be directed to the corresponding author/s.

## Ethics Statement

The studies involving human participants were reviewed and approved by The Second Xiangya Hospital of Central South University approved our experimental proposal. The patients/participants provided their written informed consent to participate in this study.

## Author Contributions

FW and KH contributed to the conception of the study, writing of the manuscript, performed the data analysis, interpretation, and discussion of the results of the analysis. XW supervised the study. All authors have read and approved the final manuscript.

## Conflict of Interest

The authors declare that the research was conducted in the absence of any commercial or financial relationships that could be construed as a potential conflict of interest.
